# BYPASS1-LIKE, A DUF793 Family Protein, Participates in Freezing Tolerance *via* the CBF Pathway in *Arabidopsis*

**DOI:** 10.3389/fpls.2019.00807

**Published:** 2019-06-26

**Authors:** Tao Chen, Jia-Hui Chen, Wei Zhang, Gang Yang, Li-Juan Yu, Dong-Ming Li, Bo Li, Hong-Mei Sheng, Hua Zhang, Li-Zhe An

**Affiliations:** ^1^ School of Life Sciences, The Key Laboratory of Cell Activities and Stress Adaptations, Ministry of Education, Lanzhou University, Lanzhou, China; ^2^ College of Life Sciences, Zhejiang University, Hangzhou, China; ^3^ School of Forestry, Beijing Forestry University, Beijing, China

**Keywords:** 14-3-3 proteins, *Arabidopsis*, CBF pathway, cold stress, DUF793, protein degradation

## Abstract

The C-REPEAT BINDING FACTOR signaling pathway is strictly modulated by numerous factors and is essential in the cold response of plants. Here, we show that the DUF793 family gene *BYPASS1-LIKE* modulates freezing tolerance through the CBFs in *Arabidopsis*. The expression of *B1L* was rapidly induced under cold treatment. Comparing to wild type, *B1L* knockout mutants were more sensitive to freezing treatment, whereas B1L-overexpressing lines were more tolerant. The expression of *CBF*s and CBF target genes was significantly decreased in *b1l* mutant. Using yeast two-hybrid screening system, 14-3-3λ was identified as one of proteins interacting with B1L. The interaction was confirmed with bimolecular fluorescence complementation assay and co-immunoprecipitation assay. Biochemical assays revealed that *b1l* mutation promoted the degradation of CBF3 compared to wild type, whereas *14-3-3κλ* mutant and *b1l 14-3-3κλ* mutant suppressed the degradation of CBF3. Consistently, *14-3-3κλ* and *b1l 14-3-3κλ* mutants showed enhanced freezing tolerance compared to wild type. These results indicate that B1L enhances the freezing tolerance of plants, at least partly through stabilizing CBF. Our findings improve our understanding of the regulation of CBF in response to cold stress.

## Introduction

Temperature is one of the most important environmental factors that affect the survival, growth, and reproduction of plants. Plants adapt to freezing stress through multiple physiological and biochemical processes. After exposure to low temperatures above freezing, temperate plants acquire freezing tolerance, a process that is termed cold acclimation (Guy, 1990; Thomashow, 1999). The expression of a class of *APETALA2/ETHYLENE-RESPONSIVE FACTOR (AP2/ERF)* transcription factor, *C-REPEAT BINDING FACTOR/DROUGHT RESPONSE ELEMENT BINDING FACTOR 1B (CBF/DREB1)*, is rapidly induced under cold stress, playing a central role in the cold acclimation of *Arabidopsis* (Stockinger et al., 1997; Liu et al., 1998; Thomashow, 1999). CBFs bind to the *CRT/DRE* element of *COLD-REGULATED (COR)* genes, inducing their expression and conferring an enhanced freezing tolerance (Yamaguchi-Shinozaki and Shinozaki, 1994; Stockinger et al., 1997; Gilmour et al., 1998; Liu et al., 1998; Thomashow, 1999).

The expression of *CBF*s is regulated by numerous transcription factors. Using *CBF3-LUC* transgenic plants, Chinnusamy et al. identified a basic helix-loop-helix (bHLH) transcription factor named INDUCER OF CBF EXPRESSION 1 (ICE1; Chinnusamy et al., 2003). ICE1 promotes the expression of *CBF3* through the binding to *MYC* cis-elements within the promoter region of *CBF3* (Chinnusamy et al., 2003). BRASSINAZOLE-RESISTANT 1 (BZR1), LATE ELONGATED HYPOCOTYL (LHY), and CALMODULIN-BINDING TRANSCRIPTION ACTIVATOR 3 (CAMTA3) were also found to positively regulate the expression of *CBF*s (Doherty et al., 2009; Dong et al., 2011; Li et al., 2017a,b). On the other hand, MYB15, ETHYLENE-INSENSITIVE 3 (EIN3), and PHYTOCHROME-INTERACTING FACTOR 3 (PIF3) repress *CBF* expression (Agarwal et al., 2006; Shi et al., 2012; Jiang et al., 2017).

The posttranslational regulation of CBF is also involved in the plant response to cold stress (Liu et al., 2017; Ding et al., 2018). In this process, 14-3-3 proteins are phosphorylated by COLD-RESPONSIVE PROTEIN KINASE 1 (CRPK1) and translocated from the cytoplasm to the nucleus, where 14-3-3 proteins can interact with CBFs and trigger the degradation of CBFs through the 26S proteasome pathway (Liu et al., 2017). By contrast, BTF3-LIKE (BTF3L) inhibits the degradation of CBFs by interacting with CBF proteins (Ding et al., 2018). However, the proteins that negatively modulate the 14-3-3λ-mediated degradation of CBF remain unknown.

In *Arabidopsis*, at least 12 proteins contain a conserved DUF793 domain, but only few members have been functionally characterized. BYPASS1 (BPS1) is required to produce a root-sourced signal that moves to the shoot and arrests growth of shoot, through modification of cytokinin signaling (Van Norman et al., 2004, 2011; Lee et al., 2016). ROH1 interacts with the exocyst subunit EXO70A1 and is involved in the localized deposition of seed coat pectin (Kulich et al., 2010). *At1g74450* gene affects the plant height, pollen development, and composition of the inner seed coat mucilage layer (Visscher et al., 2015). *AT1G18740*, which we named as BYPASS1-LIKE (B1L), and *AT1G74450* are both responsive to multiple abiotic stresses (Ma and Bohnert, 2007). However, more insight into the molecular function of the DUF793 proteins is in need.

In this study, we found that *B1L*, which is rapidly induced under cold treatment, modulates freezing tolerance through the CBFs in *Arabidopsis*. To be specific, B1L reduces the degradation of CBFs through the interaction with 14-3-3λ. Our results indicate that B1L positively modulates plant freezing tolerance, at least partly through stabilizing CBFs.

## Materials and Methods

### Plant Materials and Growth Conditions

*Arabidopsis thaliana* Col-0 was used as the wild type. The mutant and transgenic lines that were used in this study were as follows: *b1l* (SALK_019913), *14-3-3λ* (SALK_075219) (Zhou et al., 2014; Liu et al., 2017), *14-3-3κ* (SALK_148929) (Van Kleeff et al., 2014), *cbfs* (Jia et al., 2016), *b1l 14-3-3λ, 14-3-3κλ, b1l 14-3-3κλ, b1l cbfs, Super:CBF3-MYC* (Liu et al., 2017), *Super:CBF3-MYC/b1l, B1L b1l #1, B1L b1l #2*, B1L-OE #1, B1L-OE#2, *ProB1L:B1L-GFP* #1, and *ProB1L:GUS*.

*b1l* was obtained from ABRC. *14-3-3κλ* was generated by crossing *14-3-3λ* and *14-3-3κ. 14-3-3λ, 14-3-3κ*, and *14-3-3κλ* were kindly provided by the Li Jia laboratory of Lanzhou University. *cbfs* and *Super:CBF3-MYC* were kindly provided by the Shu-Hua Yang laboratory from the China Agricultural University. *b1l 14-3-3κλ, Super:CBF3-MYC/b1l*, and *b1l cbfs* were generated through genetic crossing.

The *ProB1L:B1L-3×FLAG* fusion and the *b1l* restored plants (*B1L b1l* #1 and #2) were obtained *via* amplifying the B1L genomic region, including the 2000-bp promoter fragment, and cloning the resulting PCR product into the pMDC302 Gateway binary vector. The *ProB1L:B1L-GFP* transgenic plants (*ProB1L:B1L-GF*P #1) were obtained by amplifying the same genomic region and cloning it into the pMDC107 Gateway binary vector. The *ProB1L:GUS* transgenic plants were obtained by amplifying the ProB1L fragment and cloning it into the pBIB-GUS vector. The *35S:YFP-B1L* fusion and the B1L-overexpressing transgenic lines (B1L-OE #1 and #2) were obtained by amplifying B1L cDNA and cloning the resulting PCR product into the pEarlygate104 Gateway binary vector.

Plants were grown at 22°C under long-day conditions (16 h light/8 h dark) in soil or agar plates (1/2 MS, 1% sucrose, and 0.8% agar).

All primer sequences that were used in this study are listed in Supplementary Table S1.

### Plant Freezing Assay

The plant freezing assays were performed as previously described (Zhu et al., 2004; Miura et al., 2007) with modifications. Plants were grown in soil at 21°C under long-day (LD) conditions for 3 weeks before the treatments were performed. For each line, the plant freezing assay was performed with four pots of 16 plants. For the treatments without cold acclimation, the pots with different plants were alternately placed in a controlled-temperature chamber (MIR-254; SANYO) for approximately 30 min at 0°C and then for 1 h at 0°C before the temperature was decreased by 1°C/h. The final desired sub-zero temperature was maintained for the indicated period before the temperature was again increased to 4°C. The plants were then kept at 4°C for 12 h before they were returned to 21°C. Survival was scored 5 days later, and those plants able to maintain a green color at the shoot apex were counted as survivors. For the cold acclimation experiments, 3-week-old plants were acclimated to 4°C in the light for 3 days. The freezing treatment was then performed in the same manner as that for the non-acclimated plants, with the final desired freezing temperature maintained for the indicated period. The experiments were conducted with three independent biological replicates.

### Electrolyte Leakage Assay

Electrolyte leakage of detached leaves from 3-week-old plants was measured as previously described (Zhao et al., 2017) with modifications. Plants were grown in soil at 21°C in LD conditions, and the fifth oldest leaf was used. The leaves were placed in tubes containing 100 μl of deionized water. Ice chips were added to the tubes, and the tubes were kept at 0°C for 30 min, followed by a temperature decrease of 1°C/h. The samples were removed at the indicated temperature points and immediately placed on ice. Ten milliliters of deionized water were added to each tube, and the samples were incubated for 1 h at 21°C under gentle shaking, after which the conductivity of the solution was determined *via* a conductivity meter (DDSJ-308A; INESA). The tubes were then autoclaved at 120°C for 30 min, and the conductivity of the solution was measured again after the samples were cooled to 21°C. Electrolyte leakage was quantified as a percentage of the conductivity after the treatment relative to total conductivity. The experiments were conducted with three independent biological replicates.

### qRT-PCR and RT-PCR Assays

Total RNA was extracted with a RNAprep pure plant kit (TIANGEN) and treated with DNaseI to digest the DNA. First-strand cDNA was synthesized from 1 μg of RNA using the RevertAid First Strand cDNA Synthesis Kit (Thermo Scientific) according to the manufacturer’s instructions.

For the qRT-PCR assay, 12-day-old seedlings were grown on agar plates and treated at 4°C in the light, and the plant material was collected in a time-course manner. qRT-PCRs were performed with the SsoFast EvaGreen Supermix (Bio-Rad) using the CFX96 Real-Time System (Bio-Rad). Actin2/8 was used for the normalization of the results (Shi et al., 2012; Liu et al., 2017; Li et al., 2017a). qRT-PCRs were typically performed with at least three independent biological samples, and each was measured with at least three technical repeats. The statistical significance of the differences between two samples was assessed using a Student’s *t* test.

For the RT-PCR analysis, the roots and whole seedlings were collected from 12-day-old seedlings; the leaves and stems were collected from 5-week-old plants; and the flowers and siliques were collected from 8-week-old plants. *β-TUBULIN* was used as an internal control. Ethidium bromide staining was used to detect the PCR products.

### Confocal Microscopic Analysis

B1L cDNA was amplified and cloned into the pEarleygate104 Gateway binary vector. The plasmid was introduced into *Agrobacterium tumefaciens* GV3101 and transiently expressed in *N. benthamiana* leaves. Two days after infiltration, the YFP fluorescence signal was detected with a confocal microscope (Leica SP8). The 5-day-old *ProB1L:B1L-GFP* and B1L-OE with YFP tag seedlings were also used for a subcellular localization assay.

### Histochemical GUS Reporter Gene Expression Analysis

The GUS staining assay was performed as previously described (Chen et al., 2016) with T3 *ProB1L:GUS* transgenic plants of 1-day-old seedlings, 2-day-old seedlings, 10-day-old seedlings, and 8-week-old mature plants. After staining, the samples were rinsed with acetic acid/methanol [1:3 (v/v)]. The images were collected on a stereomicroscope (Nikon SMZ800).

### Y2H Screening and Assay

AH109 was used as a host strain. B1L cDNA was subcloned into the pGBKT7 Gateway binary vector. The transcriptional activation of B1L-pGBKT7 was detected, and B1L-pGBKT7 was used as bait to screen an *Arabidopsis thaliana* cDNA library. The transformation was performed according to the Clontech Yeast Protocols Handbook (PT3024), with selection on media lacking leucine (Leu), tryptophan (Trp), histidine (His), and adenine (Ade). The positive clones were isolated and sequenced. To determine the interaction between B1L and 14-3-3λ in yeast, *B1L*-N terminal, *B1L*-C terminal, *B1L*^S213A^, and *B1L*^S213D^ were amplified and cloned into the pGBKT7 Gateway binary vector. The coding sequence of *14-3-3λ* was amplified and cloned into the pGADT7 Gateway binary vector. The coding sequence of *14-3-3ψ* was also amplified and cloned into the pGADT7 Gateway binary vector to analyze the interaction between B1L and 14-3-3ψ. The yeast transformation and growth assays were performed as described above.

### BiFC Assay

To determine the interaction between B1L and 14-3-3λ, *B1L* cDNA, *B1L^S213A^*, and *B1L^S213D^* were amplified and cloned into PNYFP-X, and 14-3-3λ cDNA was amplified and cloned into the PCCFP-X Gateway binary vector. Plasmids containing YFP^N^-B1L and YFP^C^-14-3-3λ, YFP^N^-B1L and YFP^C^, YFP^N^ and YFP^C^-14-3-3λ, YFP^N^-B1L^S213D^ and YFP^C^-14-3-3λ, or YFP^N^-B1L^S213A^ and YFP^C^-14-3-3λ were introduced into *A. tumefaciens* GV3101 and transformed to *N. benthamiana* leaves. Two days after infiltration, the YFP fluorescence signal was detected using a Leica SP8 confocal microscope.

### CoIP Assay

*35S:B1L-FLAG/35S:14-3-3λ-MYC* or *35S:FLAG/35S:14-3-3λ-MYC* were co-expressed into *N. benthamiana* leaves. Total protein was subsequently extracted in IP buffer containing 50 mM Tris–HCl, pH 7.6; 150 mM NaCl; 10% glycerol; and 1× Cocktail. The cell debris were removed *via* two 12-min centrifugations at 16,000 g at 4°C. The supernatant was collected and incubated with anti-FLAG agarose (Abmart) overnight at 4°C. After washing with IP buffer five times, the co-immunoprecipitated products were separated by SDS-PAGE and detected with anti-MYC (1:5,000, Abcam) and anti-FLAG (1:10,000, Abmart) antibodies.

### Protein Degradation Assay

Protein degradation assays were performed as previously described (Wang et al., 2009; Liu et al., 2010) with modifications. *Arabidopsis* seedlings were harvested and ground to a fine powder in liquid nitrogen. Total protein was subsequently extracted in degradation buffer containing 25 mM Tris-HCl, pH 7.5; 10 mM NaCl; 10 mM MgCl_2_; 5 mM DTT; and 1× Cocktail, and the protein concentration was determined.

For the cell-free degradation assay, 100 μg purified MBP-CBF3 recombinant proteins were incubated with the total proteins that were extracted from wild type, *b1l*, B1L-OE #1, *14-3-3*κ*λ*, or *b1l 14-3-3*κ*λ* plants in the presence of 1 mM ATP (Sigma) at 25°C for different time courses, and the CBF3-MBP proteins were detected *via* immunoblotting with an anti-MBP antibody (1:10,000; Abcam). MG132 (Sigma) was added to the various degradation assays, as indicated.

### Software Availability

Band intensity quantifications of CBF3 were performed using the ImageJ^1^. The motifs within B1L protein that are likely to be phosphorylated and bound by 14-3-3 proteins were predicted with Scansite 4^2^. The diagram for the B1L protein was performed using the IBS^3^.

## Accession Numbers

Sequence data from this article can be found in the Arabidopsis Genome Initiative or GenBank/EMBL/Swiss-Prot databases under the following accession numbers: B1L (AT1G18740), CBF1 (AT4G25490), CBF2 (AT4G25470), CBF3 (AT4G25480), COR15a (AT2G42540), COR15b (AT2G42530), COR47 (AT1G20440), RD29A (AT5G52310), ERF4(AT3G15210), ERF11(AT1G28370), 14-3-3λ (AT5G10450), 14-3-3κ (AT5G65430), and 14-3-3ψ (AT5G38480).

## Results

### Cold Induces the Expression of *B1L*, Which Positively Modulates Freezing Tolerance

**Figure 1:**
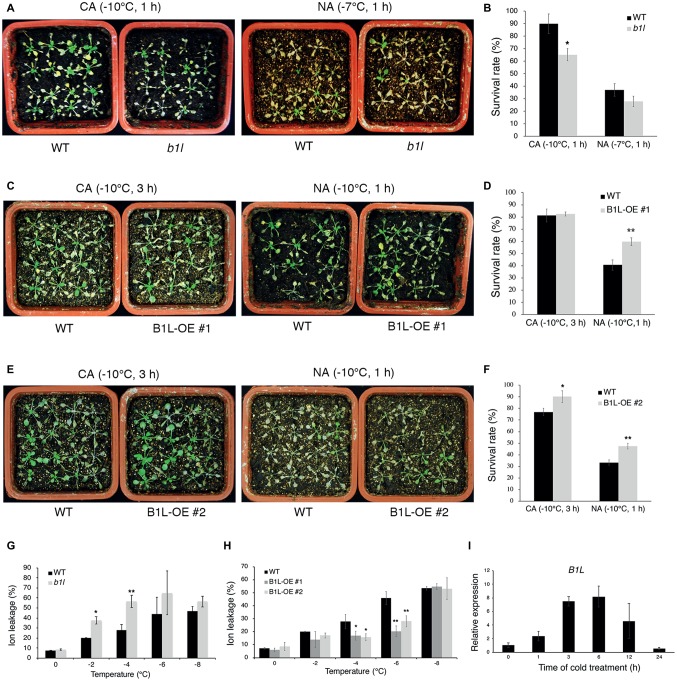
B1L positively modulates freezing tolerance in *Arabidopsis*. **(A–F)** Freezing tolerance **(A,C,E)** and survival rates **(B,D,F)** of wild type (WT), *b1l* mutant, and B1L-overexpressing plants under non-acclimated (NA) or cold-acclimated (CA) conditions. The 3-week-old plants were treated at −7 or −10°C for 1 h (NA) or were pretreated at 4°C for 3 days and then treated at −10°C for 1 or 3 h (CA). For each line, the survival rate assay was performed with four pots of 16 plants and scored 5 days later. The photos presented one pot of each line. The data are shown as means of three independent biological replicates ± SD. Asterisks indicate significant differences (*p < 0.05, and **p < 0.01) from wild type. **(G,H)** Ion leakage of wild type, *b1l* mutant, and B1L-overexpressing plants in **(A,C,E)** after exposure to the temperature indicated. Data are means ±SD. *n* = 4 leaves, each from a different plant. Asterisks indicate significant differences (*p < 0.05 and **p < 0.01) from wild type. **(I)** Expression of *B1L* in wild type under cold treatment. Total RNA was extracted from 12-day-old seedlings treated at 4°C for 0, 1, 3, 6, 12, 24 h and then subjected to qRT-PCR. *Actin2/8* was used as a control. The expression of *B1L* in untreated wild type was set to 1. The data are shown as means of three independent biological replicates ± SD.

To investigate the function of B1L in plant freezing tolerance, a T-DNA insertion mutant (*b1l*) was obtained from the Arabidopsis Biological Resource Center (ABRC), and the complete disruption of *B1L* expression in the *b1l* mutant was verified (Supplementary Figure S1A). Three-week-old plants showing similar growth and development in wild type and *b1l* were used to examine the freezing tolerance (Supplementary Figure S1B). The *b1l* mutant was more sensitive to freezing treatment than wild type under cold-acclimated (CA) conditions (Figures 1A,B). *ProB1L:B1L-3×FLAG* was then transformed to *b1l* mutant, and the complementation lines (*B1L b1l*) fully restored the freezing sensitivity of *b1l* (Supplementary Figure S2). Transgenic plants overexpressing *B1L-YFP* (B1L-OE) were also used to examine the role of B1L (Supplementary Figure S3). B1L-OE #1 and #2 plants were more freezing tolerant compared to wild type, particularly under non-acclimated (NA) conditions (Figures 1C–F). Consistently, ion leakage in the *b1l* mutant was higher than that in wild type after freezing treatment (Figure 1G), whereas the ion leakage of the B1L-OE #1 and #2 plants was lower than that of wild type after freezing treatment (Figure 1H). A quantitative real-time PCR (qRT-PCR) assay was used to investigate the expression of *B1L* under cold treatment (4°C). The expression of *B1L* quickly increased after 1 h and reached a peak after 6 h (Figure 1I). These data indicate that B1L acts as a positive regulator of freezing tolerance in *Arabidopsis*.

### B1L Was Shown to Modulate the Expression of Genes in the CBF Pathway

**Figure 2:**
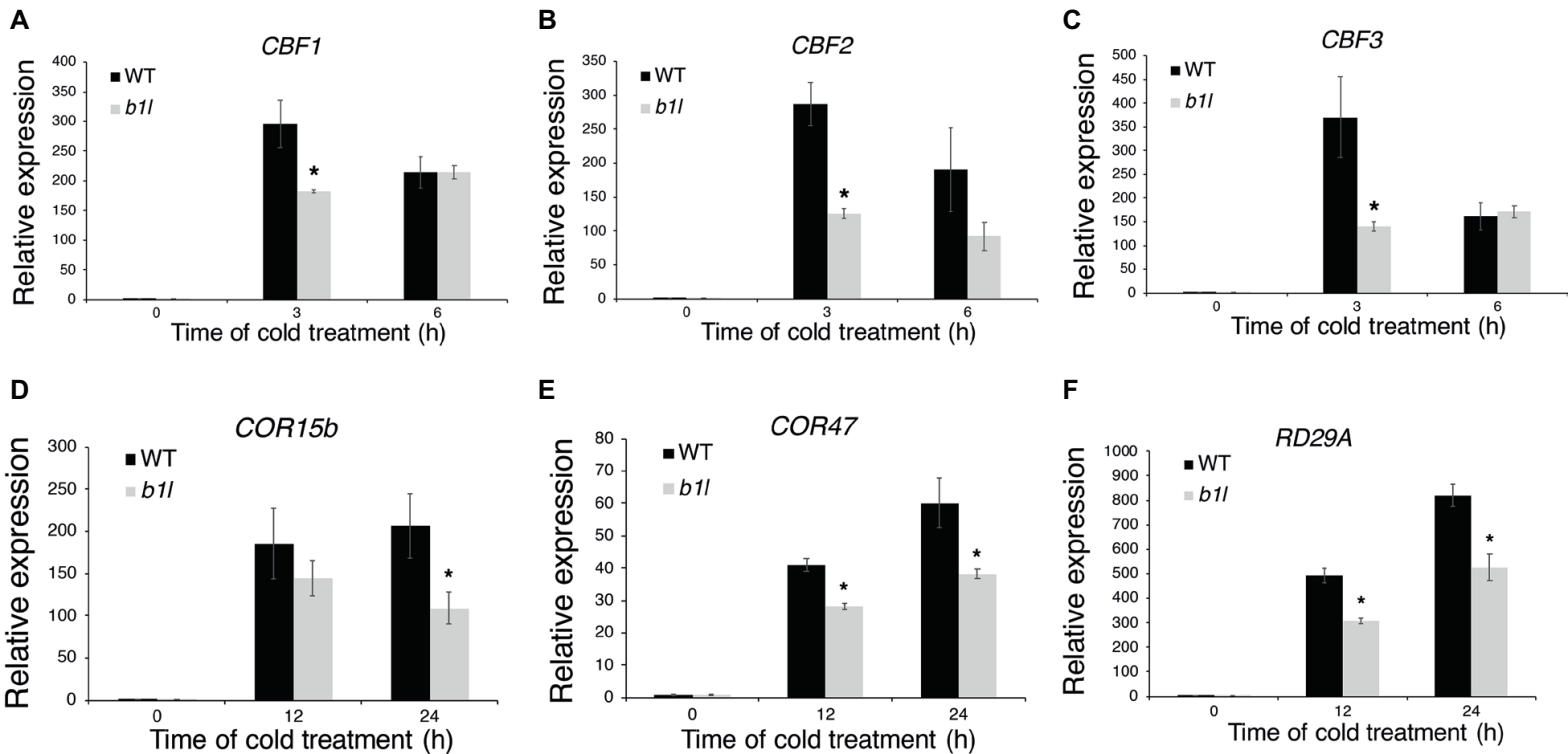
B1L is a positive regulator of CBF pathway genes. The expression of *CBF1, CBF2*, and *CBF3* **(A–C)** and CBF target genes *COR15b, COR47*, and *RD29A* **(D–F)** in wild type and *b1l* seedlings was determined *via* qRT-PCR analyses. Total RNA was extracted from 12-day-old seedlings treated at 4°C for 0, 3, 6 h for *CBF*s and 0, 12, 24 h for CBF target genes and then subjected to qRT-PCR. The data are shown as means of three independent biological replicates ± SD. Asterisks indicate significant differences (*p < 0.05) from wild type.

To examine whether B1L participates in plant cold acclimation, the expression of CBF pathway genes that perform important roles in cold acclimation was examined through qRT-PCR. The qRT-PCR results showed that both the *CBF*s and CBF target genes were dramatically reduced in the *b1l* mutants compared to wild type (Figure 2). These results indicate that B1L positively regulates the expression of CBF pathway genes.

### B1L Is Expressed in Most Tissues and B1L Protein Localizes to the Cytoplasm and the Nucleus

**Figure 3:**
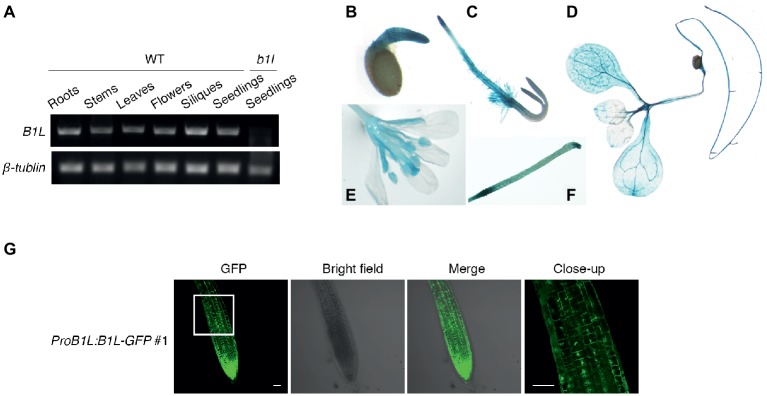
The expression of *B1L* in different tissues and the subcellular localization of B1L. **(A)** RT-PCR analysis of *B1L* transcripts in the different tissues of wild type. The *B1L* transcript in the *b1l* mutant seedlings was used as a negative control. *β-TUBULIN* was used as a loading control. **(B–F)** Histochemical analysis of the GUS reporter gene expression driven by the B1L promoter. The GUS signal was detected in 1-day-old seedlings **(B)**, 2-day-old-seedlings **(C)**, 10-day-old seedlings **(D)**, and 8-week-old mature plants **(E,F)**. **(G)** Localization of B1L in transgenic plants expressing B1L-GFP driven by its native promoter (*ProB1L:B1L-GFP* #1). The signals in the root tips of 5-day-old plants were visualized. Bar = 50 μm.

To analyze the expression pattern of *B1L*, the total RNA from different tissues of wild type was collected and tested. Semi-quantitative RT-PCR results showed that *B1L* was expressed in most tissues; the mRNA from *b1l* seedlings was used as a negative control (Figure 3A). GUS staining assay using *ProB1L:GUS* transgenic plants revealed that B1L was predominately expressed in the roots, leaves, and flowers (Figures 3B–F).

To examine the subcellular localization of B1L, A *ProB1L:B1L-GFP* construct was generated and transformed to *b1l* mutant plants (*ProB1L:B1L-GFP* #1). Under microscope, GFP fluorescence signal was detected in both the cytoplasm and nucleus (Figure 3G). The transient expression of *35S:YFP-B1L* in *Nicotiana benthamiana* and the stable expression of *35S:YFP-B1L* in *Arabidopsis* were also used to show that B1L localizes to the cytoplasm and nucleus (Supplementary Figure S4).

### B1L Directly Interacts With 14-3-3λ Both *in vitro* and *in vivo*

**Figure 4:**
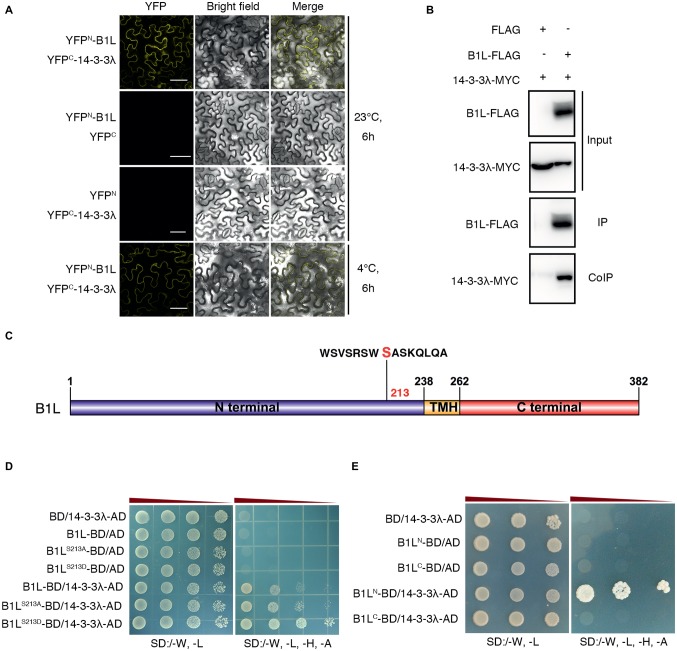
B1L interacts with 14-3-3λ, and serine 214 in the N-terminal of B1L modulates the interaction between B1L and 14-3-3λ. **(A)** BiFC analysis in *N. benthamiana* showing the interaction between B1L and 14-3-3λ. The plants were pretreated with or without 4°C for 6 h, and then, the YFP signals were detected. Bar = 50 μm. **(B)** CoIP assay showing the interaction between B1L and 14-3-3λ in plants. Total proteins were extracted from transformed*N. benthamiana* leaves, immunoprecipitated with an anti-FLAG antibody, and detected with an anti-MYC antibody and an anti-FLAG antibody. **(C)** Diagram of B1L-truncated proteins and B1L-mutated proteins used for the Y2H assays. Serine 213 in the N-terminal of B1L was a candidate phosphorylated site that can be recognized by 14-3-3 proteins. This site was mutated to alanine (B1L^S213A^) and aspartic acid (B1L^S213D^) to mimic nonphosphorylation and autophosphorylation of B1L, respectively. The N-terminal (amino acids 1-238) and the C-terminal (amino acids 262-382) of B1L were used to identify the binding domain of B1L that interacts with 14-3-3λ. **(D,E)** Y2H analysis of the interaction between 14-3-3λ and B1L native protein, B1L-mutated proteins or B1L-truncated proteins. Panels show yeast serial decimal dilutions.

To further investigate B1L function in the freezing tolerance of plants, a yeast two-hybrid (Y2H) screening system was used. A 14-3-3 family protein, 14-3-3λ, was identified. A bimolecular fluorescence complementation (BiFC) assay was used to confirm the interaction, and the results showed that B1L interacts with 14-3-3λ under both cold treatment (4°C, 6 h) and normal conditions (Figure 4A). A co-immunoprecipitation (coIP) assay using co-expressed B1L-FLAG and 14-3-3λ-MYC was also performed and the result verified that B1L directly interacts with 14-3-3λ *in vivo* (Figure 4B).

The 14-3-3 proteins are well-known to bind to many proteins that are phosphorylated by recognizing phosphoserine or phosphothreonine within their conserved binding motifs (Jaspert et al., 2011; Wang et al., 2011; Yoon and Kieber, 2013; Zhou et al., 2014; Huang et al., 2018). To search for 14-3-3 motifs in B1L sequence, the Scansite 4^4^ was used to predict the candidate site, and Serine 213 in the N-terminal of B1L was identified (Figure 4C). This site was then mutated to alanine (B1L^S213A^) and aspartic acid (B1L^S213D^) to mimic nonphosphorylation and autophosphorylation, respectively. The Y2H assays showed that both B1L^S213A^ and B1L^S213D^ could interact with 14-3-3λ (Figure 4D). The BiFC assay was also performed and showed the same results (Supplementary Figure S5). These results suggest that B1L^S213A^ and B1L^S213D^ may not be sufficient to affect the interaction between B1L and 14-3-3λ. Then, the N-terminal (amino acids 1-238) and the C-terminal (amino acids 262-382) of B1L were used to identify the binding domain of B1L that interacts with 14-3-3λ. The Y2H assays showed that the N-terminal of B1L, but not the C-terminal of B1L, is sufficient for the interaction (Figure 4E).

### B1L Regulates Freezing Tolerance, Possibly Through a 14-3-3λ-Dependent Pathway

**Figure 5:**
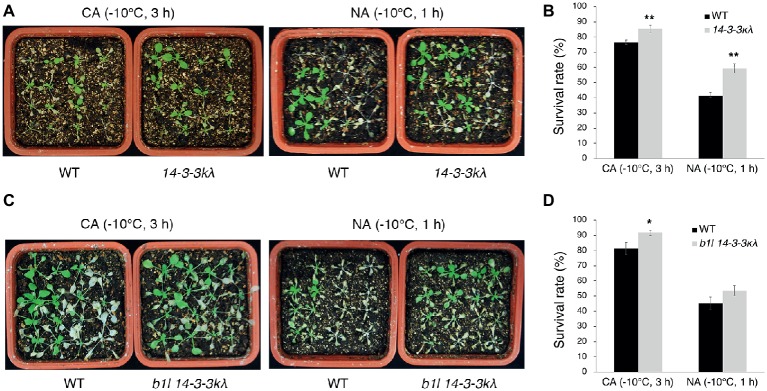
B1L regulates freezing tolerance *via* a 14-3-3λ-dependent way. Freezing tolerance **(A,C)** and survival rates **(B,D)** of *14-3-3kλ* mutants and *b1l 14-3-3kλ* mutants under NA or CA conditions. The assays were performed as in Figure 1. The data are shown as means of three independent biological replicates ± SD. Asterisks indicate significant differences (*p < 0.05, and **p < 0.01) from wild type.

**Figure 6:**
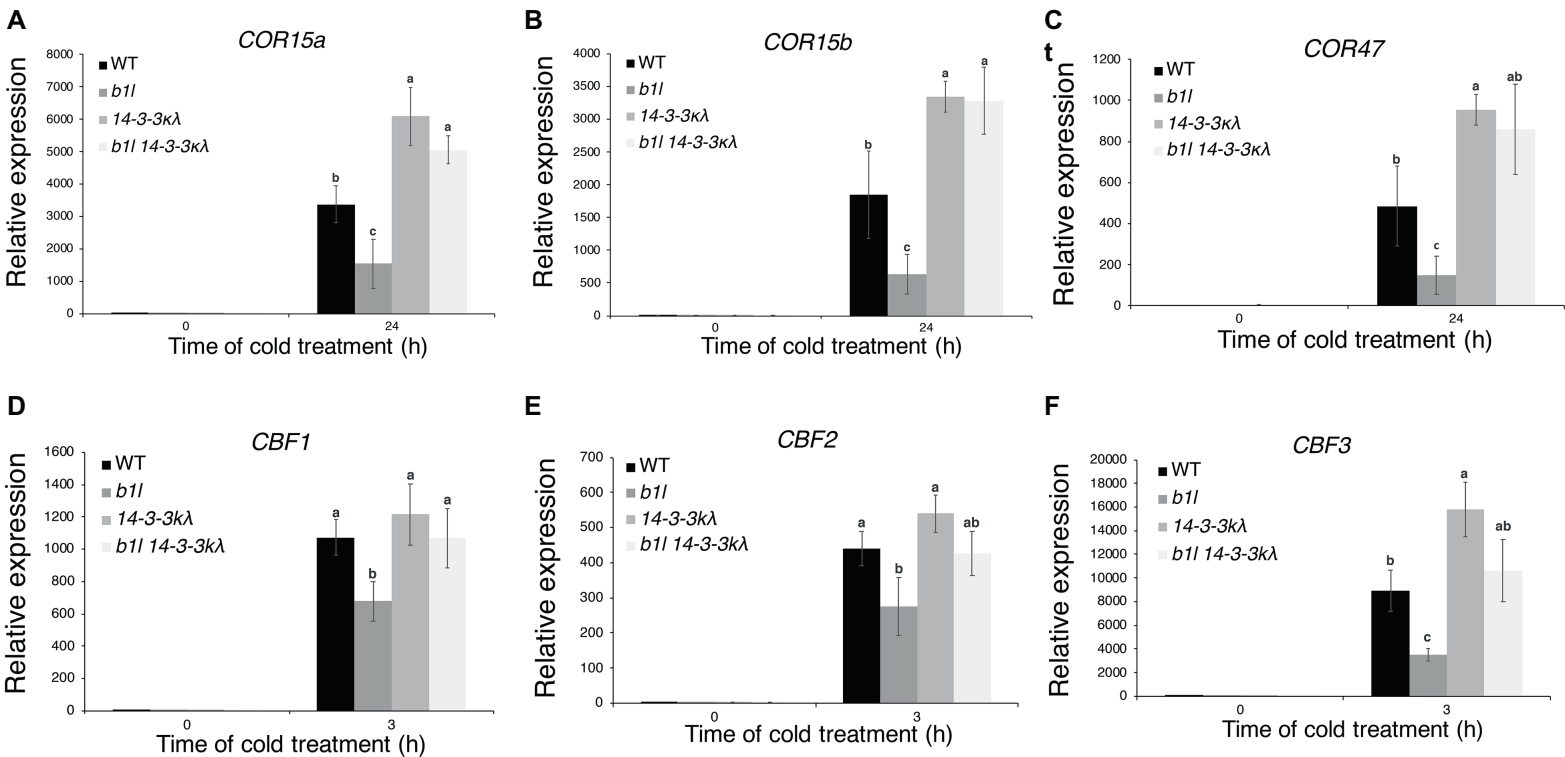
The expression of *CBF*s and *COR* genes in *14-3-3kλ* and *b1l 14-3-3kλ* under cold treatment. The expression of the CBF target genes *COR15a, COR15b*, and *COR47* **(A–C)** and *CBF* genes **(D–F)** in wild type, *b1l, 14-3-3kλ*, and *b1l 14-3-3kλ* seedlings was determined by qRT-PCR analyses. The data are shown as means of three independent biological replicates ± SD. Significant differences (*p* < 0.05) are indicated by different lowercase letters.

To understand the genetic interaction between B1L and 14-3-3λ, the *b1l 14-3-3λ* double mutant was generated. The *14-3-3λ* mutant displayed freezing tolerance similar to wild type (Supplementary Figures S6A,B), consistent with a previous report (Liu et al., 2017). The *b1l 14-3-3λ* mutant displayed a similar freezing sensitivity to *b1l* (Supplementary Figures S6C,D). 14-3-3κ is a close homologue to 14-3-3λ, and the *14-3-3κλ* double mutant showed enhanced freezing tolerance in the previous report (Liu et al., 2017), as well as in our study (Figures 5A,B). A *b1l 14-3-3κλ* triple mutant was then generated, and this mutant showed enhanced freezing tolerance compared to that in wild type (Figures 5C,D). Consistently, the expression of *COR* genes was higher in *14-3-3κλ* and *b1l 14-3-3κλ* plants than in wild type (Figures 6A–C). Intriguingly, the expression of *CBF3* was significantly higher in *14-3-3κλ* plants than in wild type (Figure 6F), which is consistent with the results from a previous study (Liu et al., 2017). qPCR results also showed that the reduced expression of all three *CBF* genes in the *b1l* mutants was rescued in *b1l 14-3-3κλ* plants (Figures 6D–F), indicating that B1L and 14-3-3 proteins may also participate in modulating the expression of *CBF*s. These results reveal that B1L regulates freezing tolerance possibly through 14-3-3λ.

### B1L Suppresses the Ubiquitin-Mediated Degradation of CBF3 *via* 14-3-3λ

The 14-3-3 proteins were shown to interact with and destabilize CBFs in the ubiquitin/26S proteasome pathway under cold stress (Liu et al., 2017). To verify whether B1L affects the degradation of CBFs, several CBF3 degradation assays were performed.

**Figure 7:**
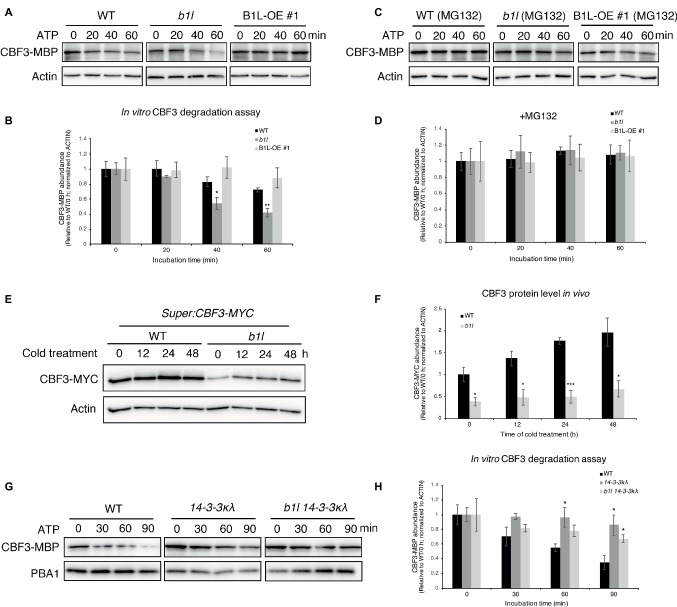
B1L inhibits the ubiquitin-mediated degradation of CBF3 *via* a 14-3-3λ-dependent way. **(A–D)** *In vitro* cell-free degradation assay showing that B1L inhibits the degradation of CBF3. The recombinant purified CBF3-MBP proteins were incubated with the total proteins extracted from wild type, *b1l* or B1L-OE #1 seedlings in the presence of ATP. These total proteins were pretreated with or without 50 μM MG132 (an inhibitor of 26S proteasome degradation) for 3 h. Typical immunoblotting results were shown **(A,C)**, along with quantification **(B,D)**. Actin was used as a control. The ratio of the band intensity of CBF3-MBP to actin without the ATP treatment was set to 1. The data are shown as means of three independent biological replicates ± SE. Asterisks indicate significant differences (*p < 0.05, and **p < 0.01) from wild type. **(E,F)** *In vivo* degradation assay showing that the CBF3 is stabilized by B1L under cold stress. *Super:CBF3-MYC* transgenic plants were crossed with *b1l* to generate *Super:CBF3-MYC/b1l* transgenic plants, and then the protein level of CBF3-MYC in both wild type background and *b1l* background was tested after different times of cold treatment. Actin served as a control. The ratio of the band intensity of CBF3 to actin without the cold treatment in *Super:CBF3-MYC* plants was set to 1. The data are shown as means of four independent biological replicates ± SE. Asterisks indicate significant differences (*p < 0.05, and ***p < 0.001) from *Super:CBF3-MYC* plants. **(G,H)** *In vitro* cell-free degradation assays showing that B1L inhibits the degradation of CBF3 *via* 14-3-3λ. The recombinant purified CBF3-MBP proteins were incubated with the total proteins extracted from wild type, *14-3-3λ* or *b1l 14-3-3λ* seedlings in the presence of ATP. PBA1 was used as a control. The ratio of the band intensity of CBF3-MBP to actin without the ATP treatment was set to 1. The data are shown as means of three independent biological replicates ± SE. Asterisks indicate significant differences (*p < 0.05) from wild type.

First, an *in vitro* cell-free degradation assay was performed using purified CBF3-MBP proteins that were expressed in *Escherichia coli*. Western blotting results showed that the degradation of the CBF3-MBP increased when it was treated with the *b1l* mutant total proteins; however, that the degradation of the CBF3-MBP was suppressed when it was treated with B1L-OE #1 total proteins (Figures 7A,B). The degradation could be inhibited by MG132 (an inhibitor of 26S proteasome degradation) (Figures 7C,D). These results indicate that the stability of CBF3 is mediated by B1L.

Then, the protein level of CBF3 *in vivo* was examined. *Super:CBF3-MYC* transgenic plants (Liu et al., 2017) were crossed with *b1l*, and the protein levels of CBF3-MYC in both wild type background and *b1l* background were analyzed after different time of cold treatment. The protein level of CBF3-MYC was much lower in *b1l* mutants than wild type before and after cold treatment (4°C) (Figures 7E,F). These results further reveal that the stability of CBF3 is mediated by B1L.

To explore the role of 14-3-3 proteins in the B1L-mediated degradation of CBFs, the purified CBF3-MBP proteins were used to test the stability of CBF3 in *14-3-3κλ* and *b1l 14-3-3κλ* mutants. Western blotting results showed that the *14-3-3κλ* mutant suppressed the degradation of CBF3-MBP compared to wild type (Figures 7G,H), as previously reported (Liu et al., 2017), and that the *b1l 14-3-3κλ* mutant also inhibited the degradation of the CBF3 (Figures 7G,H). These results show that B1L regulates CBF3 degradation *via* 14-3-3λ.

### B1L Acts Upstream of CBFs, Positively Regulating Plant Freezing Tolerance

**Figure 8:**
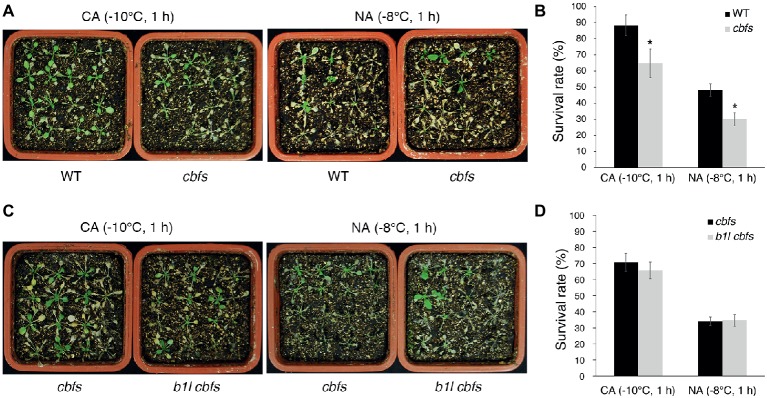
B1L acts upstream of CBFs to regulate cold signaling. Freezing tolerance **(A,C)** and survival rates **(B,D)** of *cbfs* mutants and *b1l cbfs* mutants under NA or CA conditions. The assays were performed as in Figure 1. The data are shown as means of three independent biological replicates ± SD. Asterisks indicate significant differences (*p < 0.05) from wild type.

To further examine the relationship between B1L and CBFs, *b1l* mutant was crossed with *cbfs* triple mutant (Jia et al., 2016). The *cbfs* mutant was more sensitive to freezing treatment than wild type (Figures 8A,B), consistent with previous studies (Jia et al., 2016; Liu et al., 2017). The *b1l cbfs* quadruple mutant did not aggravate the freezing sensitivity of *cbfs* (Figures 8C,D). These results further indicate that B1L positively regulating plant freezing tolerance *via* CBF proteins.

## Discussion

**Figure 9:**
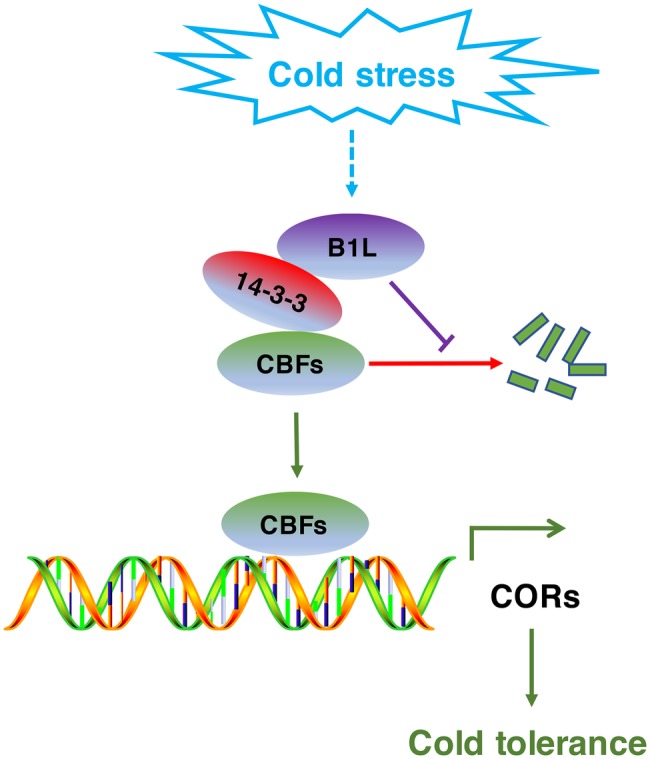
Model for the regulation of the CBF signaling pathway by B1L under cold stress. The expression of *B1L* is induced by cold stress. Then, B1L interacts with 14-3-3λ to reduce the 14-3-3λ-mediated degradation of CBFs. As a result, CBFs induce the expression of their target genes such as *COR15a, COR47*, and *RD29A* to enhance plant freezing tolerance.

CBF signaling pathway has important roles in cold acclimation. The expression of *CBF*s is regulated by numerous transcription factors. However, studies about the posttranslational regulation of CBFs are limited. Here, we show that B1L participates in regulating freezing tolerance partly through repressing the degradation of CBFs (Figure 9). Several lines of evidence were provided: (1) B1L promotes the expression of CBF pathway genes and therefore freezing tolerance in *Arabidopsis*. (2) B1L directly interacts with 14-3-3λ. (3) B1L inhibits the 14-3-3 protein-mediated degradation of CBFs. (4) The *b1l cbfs* quadruple mutant displayed a freezing sensitivity similar to *cbfs*.

The 14-3-3 proteins serve important roles in many processes, such as stomata movements, phytohormone regulation, biotic stress, and abiotic stress (Cotelle et al., 2000; Gampala et al., 2007; Wang et al., 2011; de Boer et al., 2013; Catala et al., 2014; Kaundal et al., 2017; Keicher et al., 2017; Liu et al., 2017). These proteins mostly interact with phosphorylated proteins and affect the subcellular localization, protein stability, enzymatic activity of target proteins or the interaction between the target proteins and other proteins (Jaspert et al., 2011; Wang et al., 2011; Yoon and Kieber, 2013; Zhou et al., 2014; Huang et al., 2018). Our results showed that B1L interacted with 14-3-3λ (Figure 4), indicating that B1L may also be a phosphorylated protein. Serine 213 in the N-terminal of B1L is a potential phosphorylated site that can be recognized by 14-3-3 proteins (Figure 4C), and the N-terminal of B1L is sufficient for the interaction between B1L and 14-3-3λ. However, mutating Serine 213 to alanine (B1L^S213A^) did not abolish the interaction between B1L and 14-3-3λ, indicating the possible involvement of new sites to affect the interaction between B1L and 14-3-3λ (Figure 4D; Supplementary Figure S5). The phosphorylated site of B1L that affects the interaction between B1L and 14-3-3λ and the biological roles of these sites need to be further investigated.

The 14-3-3 protein 14-3-3ψ (also known as RARE COLD INDUCIBLE 1A or GRF3) has also been shown to participate in the regulation of freezing tolerance *via* an ethylene biosynthesis pathway (Catala et al., 2014). It interacts with ACS6 and inhibits the expression of ethylene-related genes and *CBF*s, leading to decreased freezing tolerance (Catala et al., 2014). Although the expression of *CBF*s was reduced in *b1l* mutants under cold treatment (Figure 2), the expression of ethylene-related genes (*ERF4* and *ERF11*) was not significantly changed in *b1l* mutants compare to wild type (Supplementary Figures S7A,B). The interaction between B1L and 14-3-3ψ was also not observed in the Y2H system (Supplementary Figure S7C). These results indicate that B1L may specifically interact with 14-3-3λ, modulating freezing tolerance *via* an ethylene-independent pathway.

The stability of CBFs has been shown to be modulated by 14-3-3 proteins and BTF3L *via* a ubiquitin/26S proteasome pathway (Liu et al., 2017; Ding et al., 2018). HOS15, which is an E3 ligase, interacts with CBFs, affecting the expression of *COR* genes but does not participate in the regulation of CBF degradation (Park et al., 2018). Therefore, the components of the ubiquitin/26S proteasome that participate in degrading CBFs are still unknown. More proteins that are involved in the ubiquitin/26S proteasome pathway need to be identified to elucidate the mechanism of CBF stability. Our protein degradation assay *in vivo* and *in vitro* both showed that B1L affects the stability of CBF (Figures 7A–D), indicating that B1L is another regulator that participate in the regulation of CBF proteins.

The overexpression of *CBF*s leads to dwarfism and late-flowering (Jaglo-Ottosen, 1998; Achard et al., 2008; Zhou et al., 2017). ICE1 is a positive regulator of *CBF*s, and its overexpressing lines result in late-flowering, due to ICE1 directly binding to the promoter of *FLC* and inducing the expression of the *FLC* (Lee et al., 2015). Therefore, plants fine-tune CBF signaling pathway to avoid harmful effects during their development. To be specific, 14-3-3λ can be translocated to the nucleus under cold conditions, reducing the abundance of CBFs (Liu et al., 2017). Cold treatment induces the expression of *B1L* but does not obviously change the subcellular localization of B1L (Supplementary Figure S8). We hypothesis that the interaction between 14-3-3λ and B1L may lead to a delay in the nuclear translocation of 14-3-3λ after cold treatment. This translocation delay in turn allows plants to fine-tune the abundance of CBFs to adapt to cold stress.

## Data Availability

All datasets for this study are included in the manuscript and/or the Supplementary Files.

## Author Contributions

TC, J-HC, WZ, GY, and L-JY performed the experiments. TC analyzed the data. TC, HZ, and L-ZA designed the project and drafted the manuscript. D-ML, BL, and H-MS revised the manuscript critically for important intellectual content. HZ and L-ZA supervised the project and complemented the writing.

### Conflict of Interest Statement

The authors declare that the research was conducted in the absence of any commercial or financial relationships that could be construed as a potential conflict of interest.

## Footnotes

^1^https://imagej.nih.gov/ij/

^2^http://scansite4.mit.edu

^3^http://ibs.biocuckoo.org/download.php

^4^https://scansite4.mit.edu/4.0/
