# Supplementary material for: BYPASS1-LIKE, A DUF793 Family Protein, Participates in Freezing Tolerance via the CBF Pathway in Arabidopsis
Source: Front Plant Sci. 2019 Jun 26;10:807. doi: 10.3389/fpls.2019.00807 (PMC6607965; doi:10.3389/fpls.2019.00807)
Supplement: Supplementary file 1 [file Data_Sheet_1.docx]

Supplementary Material


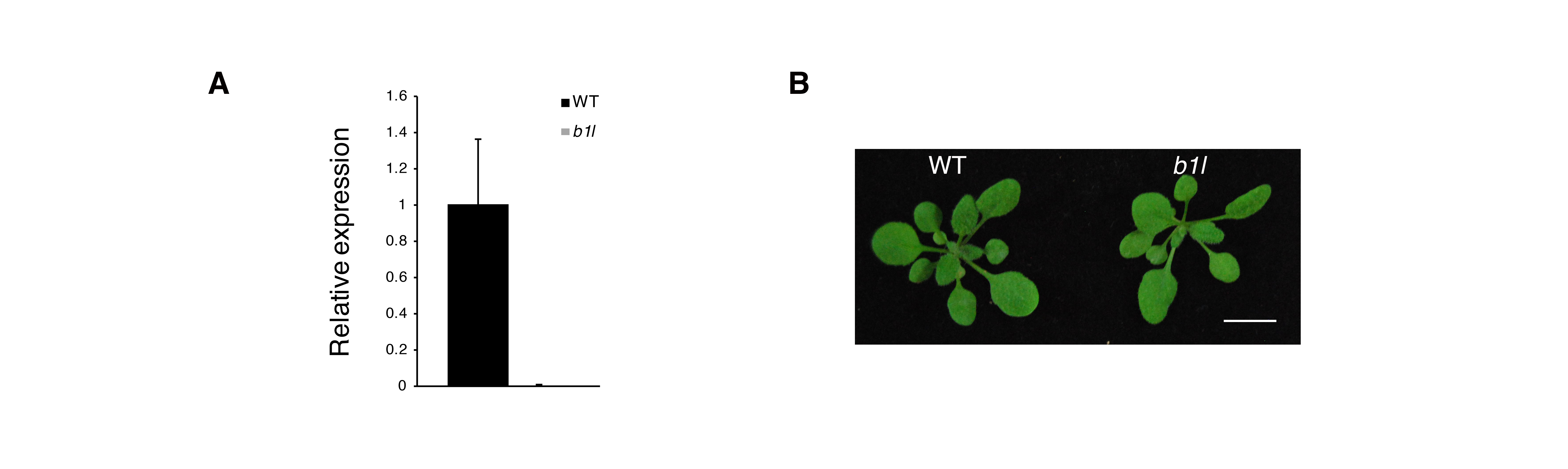


**Supplementary Figure 1.** *B**1L* expression and phenotypes of *b1l* mutants that were used in Figure 1. **(A)** The expression of *B1L* in wild type (WT) and *b1l* mutants. Total RNA was extracted from 3-week-old plants and then subjected to qRT-PCR. *Actin2*/*8* was used as a control. The expression of *B1L* in wild-type was set to 1. The data is shown as means of three independent biological replicates ±SD. **(B)** The phenotypes of 3-week-old *b1l* mutant plants. Bar = 1 cm.

**
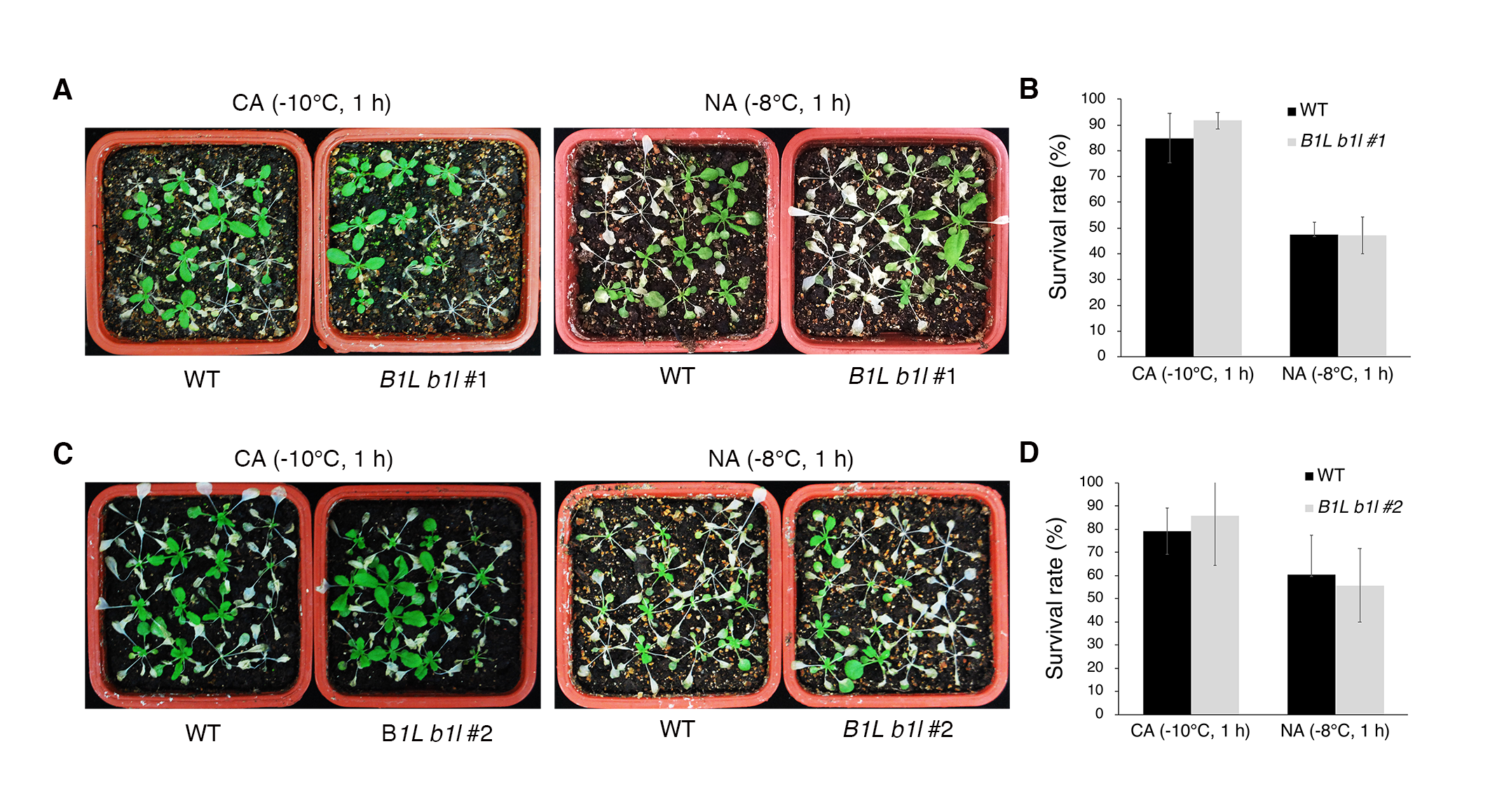
**

**Supplementary Figure 2.** Complemented lines with B1L driven by its native promoter (*B1L* *b1l*) rescue the freezing-sensitive phenotypes of *b1l.* Freezing tolerance **(A, C)** and survival rates **(B, D)** of wild type and B1L complemented lines *B1L b1l* #1 and #2 under non-acclimated (NA) or cold-acclimated (CA) conditions. The 3-week-old plants were treated at -8°C for 1 h (NA) or were pretreated at 4°C for 3 days and then treated at -10°C for 1 h (CA). For each line, the survival rate assay was performed with 4 pots of 16 plants and scored five days later. The photos presented one pot of each line. The data are shown as means of three independent biological replicates ± SD.


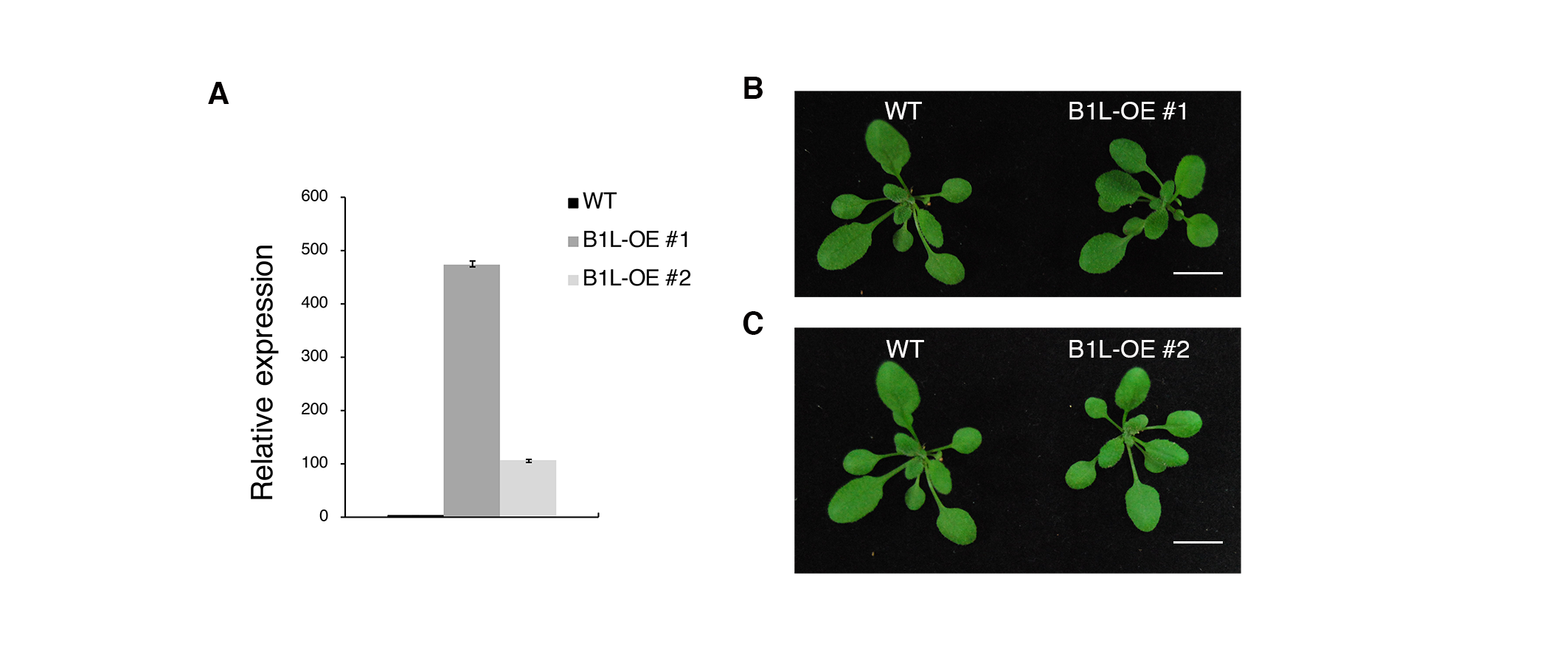


**Supplementary Figure 3.** *B1L* expression and phenotypes of B1L-overexpressing plants that were used in Figure 1. **(A)** The expression of *B1L* in wild type and B1L-overexpressing transgenic lines B1L-OE #1 and #2. The assays were performed as in figure S1A. **(B, C)** The phenotypes of 3-week-old B1L-overexpressing transgenic lines B1L-OE #1 and #2. Bar = 1 cm.


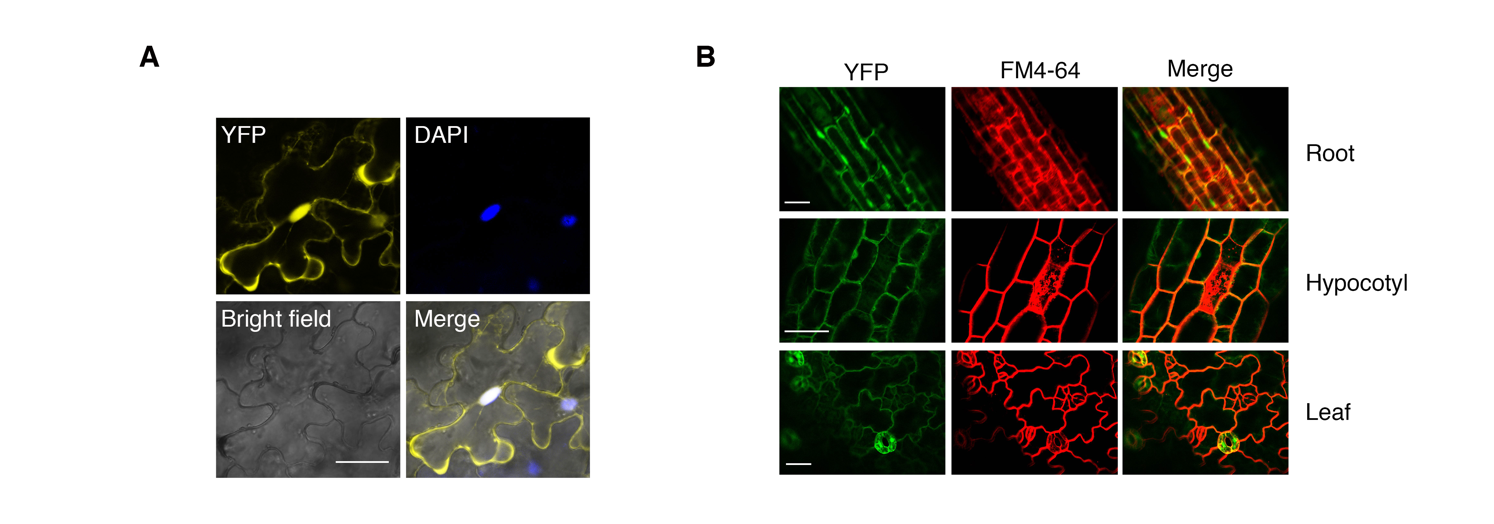


**Supplementary Figure 4.** *35S:B1L-YFP* expressed in *Nicotiana benthamiana* and *Arabidopsis thaliana* shows the subcellular localization of B1L. **(A)** *35S:B1L-YFP* transiently expressed in *N. benthamiana* leaves. **(B)** *35S:B1L-YFP* expressed in the root elongation zone, hypocotyl, and leaf epidermis of 5-day-old *A. thaliana* (B1L-OE #1). Bar = 50 μm.

**
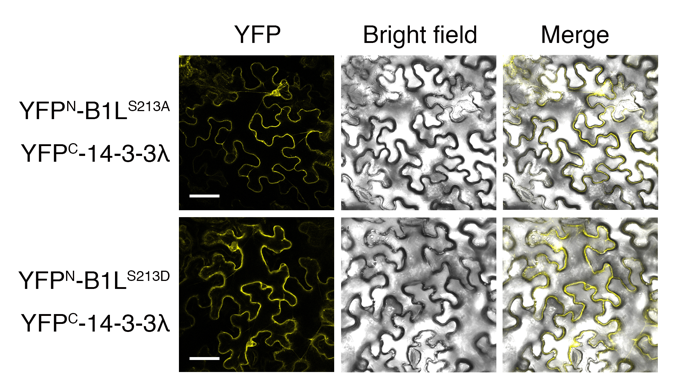
**

**Supplementary Figure 5.** 14-3-3λ interacts with B1L^S213A^ and B1L^S213D^ in a bi-molecular fluorescence complementation (BiFC) assay. Plasmids containing *Pro35S:YFP^N^-B1L^S213A^* and *Pro35S:YFP^C^-*14-3-3λ or *Pro35S:YFP^N^-B1L^S213D^* and *Pro35S:YFP^C^-*14-3-3λ were transiently cotransformed into *N. benthamiana* leaves. The YFP signals were detected 36 h later. Bar = 50 μm.


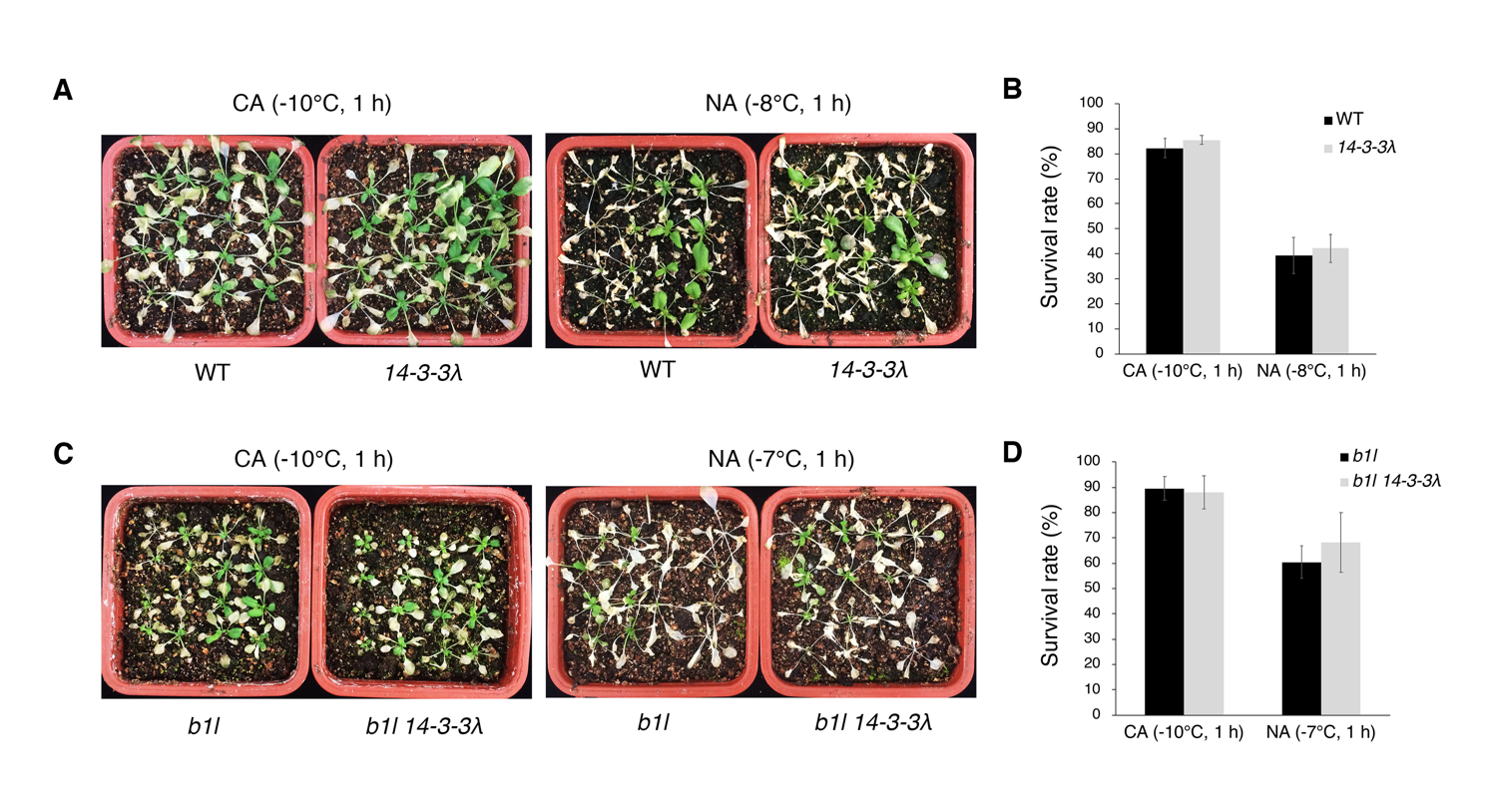


**Supplementary Figure 6.** *14-3-3λ* mutants do not show any significant freezing tolerance. Freezing tolerance **(A, C)** and survival rates **(B, D)** of *14-3-3λ* and *b1l* *14-3-3λ* mutants under non-acclimated (NA) condition or cold-acclimated (CA) condition. The assays were performed as in Figure S2.

**
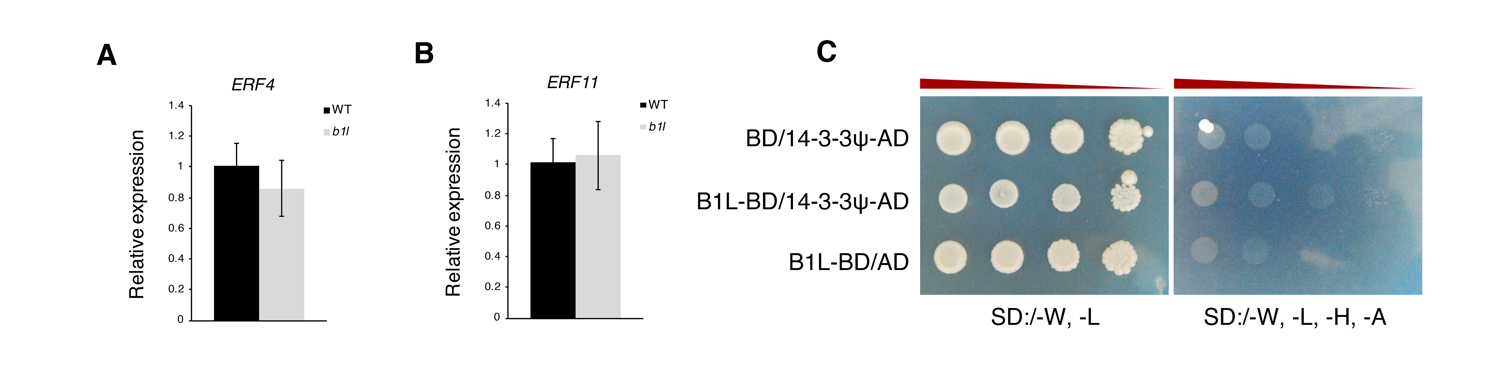
**

**Supplementary Figure 7.** B1L does not participate in the ethylene biosynthesis pathway. **(A, B)** The expression of ethylene response genes *ERF4* and *ERF11* in wild type and *b1l* mutant. 12-day-old seedlings that were grown at 23°C were used for analysis. The assays were performed as in Figure S1 A. **(C)** Yeast two hybrid assay showing that B1L does not interact with 14-3-3ψ.

**
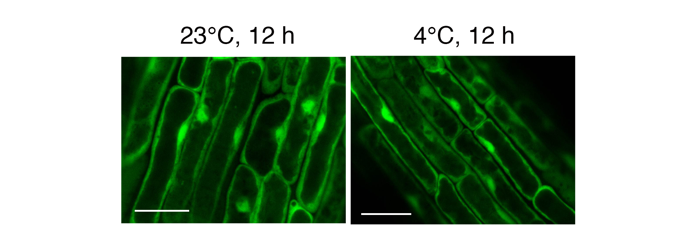
**

**Supplementary Figure 8.** The similar subcellular localization of B1L under cold treatment and normal conditions. The 5-day-old transgenic plants expressing *B1L-GFP* driven by its native promoter (*ProB1L:B1L-GFP* #1) were pretreated with or without 12 hours at 4°C, and the signals in root elongation zone were visualized under confocal microscopy. Bar = 50 μm.

| **Table S1** Oligonucleotide sequences of primers used in this study | |
| --- | --- |
| Primers used for Mutant genotyping | |
| Name | Sequence 5'-3' |
| *b1l*-LP | AGCCAGCAGAAGATTGTGAAG |
| *b1l*-RP | TCGTATTCAACCACCGATCTC |
| *14-3-3λ*-LP | TTTGAGCAATATGGG |
| *14-3-3λ*-RP | TTCCGTTGCTCTCTGGTAAC |
| *14-3-3κ*-LP | TCGAGGAACTCACAAACCAAG |
| *14-3-3κ*-RP | AGGCTTGGTTTGGCTCTTAAC |
| LB1 | GCGTGGACCGCTTGCTGCAACT |
| LBb1.3 | ATTTTGCCGATTTCGGAAC |
| Primers used for *B1L* plasmids constructing | |
| Name | Sequence 5'-3' |
| B1L-CDS-F | ATGCCAGCTACGGATTTTCAAG |
| B1L-CDS-R | AAGAGAATCAAGACTCTCAGTTCT |
| ProB1L-F | TCTTTGTTGTCTTGTGT |
| ProB1L-R | CCTCAAAACAGATGCAAAGTGG |
| B1L^N^ -R (amino acids 1-238) | ACCATTGCTAGCAACGACATCGT |
| B1L^C^ -F (amino acids 262-382) | CCTTGTCAAGACCGTGGATTGC |
| B1L^S213A^-R | TTAGAAGCTGCCCATGATCTTGAAACACTCC |
| B1L^S213A^-F | GATCATGGGCAGCTTCTAAGCAGTTACAAGC |
| B1L^S213D^-R | TTAGAAGCGTCCCATGATCTTGAAACACTCC |
| B1L^S213D^-F | GATCATGGGACGCTTCTAAGCAGTTACAAGC |
| Primers used for other genes plasmids constructing | |
| Name | Sequence 5'-3' |
| 14-3-3λ-CDS-F | ATGGCGGCGACATTAGGCAG |
| 14-3-3λ-CDS-R | CATAGAGTAGTAATAACTCAGCACACACG |
| 14-3-3ε-CDS-F | ATGGCGACGACCTTAAGCAG |
| 14-3-3ε-CDS-R | ATATGCGAGTTTCTGATGATGCAAT |
| CBF3-CDS-F | ATGAACTCATTTTCTGCTTTTTCTG |
| CBF3-CDS-R | ATAACTCCATAACGATACGTCG |
| Primers used for qRT-PCR | |
| Name | Sequence 5'-3' |
| B1L-qPCR-F | TTTCCCGTTGAATGATGACAAGG |
| B1L-qPCR-R | GTTCTGCTTCTCACAATCCGATGG |
| CBF1-qPCR-F | GCATGTCTCAACTTCGCTGA |
| CBF1-qPCR-R | ATCGTCTCCTCCATGTCCAG |
| CBF2-qPCR-F | TGACGTGTCCTTATGGAGCTA |
| CBF2-qPCR-R | CTGCACTCAAAAACATTTGCA |
| CBF3-qPCR-F | GATGACGACGTATCGTTATGGA |
| CBF3-qPCR-R | TACACTCGTTTCTCAGTTTTACAAAC |
| COR15a-qPCR-F | GGCGTATGTGGAGGAGAAAG |
| COR15a-qPCR-R | CCCTACTTTGTGGCATCCTTAG |
| COR15b-qPCR-F | AAAGCAGAGTGGTGTTGGTACCGT |
| COR15b-qPCR-R | TCATCGAGGATGTTGCCGTCACTT |
| COR47-qPCR-F | CAGTGTCGGAGAGTGTGGTG |
| COR47-qPCR-R | ACAGCTGGTGAATCCTCTGC |
| RD29A-qPCR-F | GCCGAGAAACTTCAGATTGG |
| RD29A-qPCR-R | CCATTCCTCCTCCTCCTTTC |
| ERF4-qPCR-F | TTTTGGACCTGATGGGGATCGGTA |
| ERF4-qPCR-R | GCGATCTAAACGCCGATGTCACAG |
| ERF11-qPCR-F | GCCGCTCGTGCCTACGACAA |
| ERF11-qPCR-R | CCACGGTGCTGCTCTGGCTC |
| ACTIN2/8-qPCR-F | GGTAACATTGTGCTCAGTGGTGG |
| ACTIN2/8-qPCR-R | AACGACCTTAATCTTCATGCTGC |
| Primers used for RT-PCR | |
| Name | Sequence 5'-3' |
| B1L-RT-PCR-F | TTTCCCGTTGAATGATGACAAGG |
| B1L-RT-PCR-R | GTTCTGCTTCTCACAATCCGATGG |
| β-TUBLIN-RT-PCR-F | CGTGGATCACAGCAATACAGAGCC |
| β-TUBLIN-RT-PCR-R | CCTCCTGCACTTCCACTTCGTCTTC |
